# The Collaborative Lithium Trials (CoLT): specific aims, methods, and implementation

**DOI:** 10.1186/1753-2000-2-21

**Published:** 2008-08-12

**Authors:** Robert L Findling, Jean A Frazier, Vivian Kafantaris, Robert Kowatch, Jon McClellan, Mani Pavuluri, Linmarie Sikich, Stefanie Hlastala, Stephen R Hooper, Christine A Demeter, Denise Bedoya, Bernard Brownstein, Perdita Taylor-Zapata

**Affiliations:** 1 Department of Psychiatry, University Hospitals Case Medical Center/Case Western Reserve University, Cleveland, OH, USA; 2 Cambridge Health Alliance and Department of Psychiatry, Harvard Medical School, Cambridge, MA , USA; 3 The Feinstein Institute for Medical Research of the North Shore—Long Island Health System, Manhasset, NY, USA; 4 Division of Psychiatry, Cincinnati Children’s Hospital, Cincinnati, OH, USA; 5 Department of Psychiatry, University of Washington, Seattle, WA, USA; 6 Department of Psychiatry, University of Illinois at Chicago, Chicago, IL, USA; 7 Department of Psychiatry, University of North Carolina at Chapel Hill, Chapel Hill, NC, USA; 8 Clinical Center for the Study of Development and Learning of the Carolina Institute of Developmental Disabilities, University of North Carolina at Chapel Hill, Chapel Hill, NC, USA; 9 Best Pharmaceuticals for Children Act-Coordinating Center, Premier Research, Philadelphia, PA, USA; 10 Eunice Kennedy Shriver National Institute of Child Health and Human Development, Bethesda, MD, USA

## Abstract

**Background:**

Lithium is a benchmark treatment for bipolar illness in adults. However, there has been relatively little methodologically stringent research regarding the use of lithium in youth suffering from bipolarity.

**Methods:**

Under the auspices of the Best Pharmaceuticals for Children Act (BPCA), a Written Request (WR) pertaining to the study of lithium in pediatric mania was issued by the United States Food and Drug Administration (FDA) to the National Institute of Child Health and Human Development (NICHD) in 2004. Accordingly, the NICHD issued a Request for Proposals (RFP) soliciting submissions to pursue this research. Subsequently, the NICHD awarded a contract to a group of investigators in order to conduct these studies.

**Results:**

The Collaborative Lithium Trials (CoLT) investigators, the BPCA-Coordinating Center, and the NICHD developed protocols to provide data that will: (1) establish evidence-based dosing strategies for lithium; (2) characterize the pharmacokinetics and biodisposition of lithium; (3) examine the acute efficacy of lithium in pediatric bipolarity; (4) investigate the long-term effectiveness of lithium treatment; and (5) characterize the short- and long-term safety of lithium. By undertaking two multi-phase trials rather than multiple single-phase studies (as was described in the WR), the feasibility of the research to be undertaken was enhanced while ensuring all the data outlined in the WR would be obtained. The first study consists of: (1) an 8-week open-label, randomized, escalating dose Pharmacokinetic Phase; (2) a 16-week Long-Term Effectiveness Phase; (3) a 28-week double-blind Discontinuation Phase; and (4) an 8-week open-label Restabilization Phase. The second study consists of: (1) an 8-week, double-blind, parallel-group, placebo-controlled Efficacy Phase; (2) an open-label Long-Term Effectiveness lasting either 16 or 24 weeks (depending upon blinded treatment assignment during the Efficacy Phase); (3) a 28-week double-blind Discontinuation Phase; and (4) an 8-week open-label Restabilization Phase. In December of 2006, enrollment into the first of these studies began across seven sites.

**Conclusion:**

These innovative studies will not only provide data to inform the labeling of lithium in children and adolescents with bipolar disorder, but will also enhance clinical decision-making regarding the use of lithium treatment in pediatric bipolar illness.

**Trial Registration:**

NCT00442039

## Background

In January of 2002, the United States Congress passed the Best Pharmaceuticals for Children Act (BPCA) [1] into law with the intent of improving the safety and efficacy of medications in pediatric populations. Ultimately, the initial goal of the BPCA was to establish a process for studying on-patent and off-patent drugs for use in pediatric populations. The legislation also calls for the scientific investigation of pediatric therapeutics through the conducting of pediatric studies and research to learn more about the efficacy and safety of medications in children. This occurs through a partnership of the National Institutes of Health (NIH) and the Food and Drug Administration (FDA) [2]. The Director of the NIH has delegated the authority to implement the drug development program to the Director of the Eunice Kennedy Shriver National Institute of Child Health and Human Development (NICHD), and the NICHD administers the research program through the Obstetric and Pediatric Pharmacology Branch of the Center for Research for Mothers and Children, working in cooperation with the other NIH Institutes and Centers with other significant pediatric research portfolios.

To identify off-patent medications in need of further study, the BPCA asks the NICHD, in consultation with the FDA and experts in pediatric drug development, to develop a process for prioritizing needs in pediatric therapeutics by publishing a priority list. As a result, the U.S. FDA and NICHD began collaborations to identify and prioritize medications that were to be studied in pediatric populations [2]. In 2003, the first list of drugs for which pediatric studies were needed was generated. It consisted of 20 medications, including lithium [2].

Lithium is a benchmark treatment for adult patients with bipolar disorder (BD). Lithium has been found to be efficacious in alleviating acute mania and preventing manic and mixed mood relapses in adults [3-6]. As a result, lithium is indicated in the United States for the acute and maintenance treatment of mania in BD in adults. Unfortunately, definitive randomized controlled trials of lithium have not been performed in pediatric populations that would lead to labeling of lithium for children and/or adolescents suffering from mania or mixed states in BD.

Despite the paucity of data, it should be noted that preliminary studies have found that open label treatment with lithium may be effective in the treatment of children and adolescents with bipolar disorders [7,8]. In addition, lithium is a recommended treatment for manic or mixed states for youth with BD according to published treatment guidelines for pediatric BD [9]. A major consideration regarding the study of lithium as a treatment for youths suffering from BD is the untested assumption that lithium dosing procedures and therapeutic drug level monitoring that are used in adults are applicable to children and adolescents.

## Methods

In an effort to better characterize lithium's use and efficacy in children as well as develop pediatric labeling under the auspices of the BPCA, a Written Request (WR) pertaining to lithium was issued by the FDA to the NICHD in 2004. A Written Request is a letter issued by FDA to the holder of the New Drug Application (NDA) that outlines how a pediatric study should be conducted and includes the study population, numbers of patients, study design, outcome measures, format, and time line of submission.

The study design outlined in the WR was informed by recommendations included in a published consensus paper regarding the study of mania in pediatric patients [10]. The WR for lithium noted that three studies should be executed in children and adolescents ages 7–17 years with acute mania in order to inform the labeling of lithium for this population. These three studies included a Pediatric Pharmacokinetic and Tolerability study, a Pediatric Efficacy and Safety study, and a Pediatric Long-term Safety study.

In the Pediatric Pharmacokinetic and Tolerability study, the pharmacokinetics of lithium would be examined. The WR mandated that at least 18 pediatric patients (9 males and 9 females) be enrolled in this study. In addition, an evidence-based dosing paradigm would be developed that would achieve target serum levels but also minimize toxicity. Moreover, the dosing schedule results from this study would then be utilized in the subsequent Efficacy and Long-term Safety treatment trials.

According to the WR, following the completion of the Pediatric Pharmacokinetic and Tolerability study, the Pediatric Efficacy and Safety study was to be initiated. This trial would last for a minimum of 6 to 8 weeks, and would consist of a randomized, double-blind, parallel-group, placebo-controlled acute study. As directed by the WR, this study would have a sufficient number of male and female patients to detect a difference between lithium and placebo, equivalent to the median effect size seen in adult trials. After this second trial was completed, the Pediatric Long-term Safety Study would commence so that long-term safety data could be collected. It was required that at least 100 patients be exposed to lithium for no less than 6 months for this study. Specific areas of attention for both the Efficacy and Long-term studies included safety assessments with special emphasis being placed on the examination of putative short- and long-term effects of lithium on cognition, growth, thyroid, and renal function.

Accordingly, the NICHD issued a Request for Proposals (RFP) on February 10, 2005 soliciting submissions for the study of lithium as described in the WR. The RFP indicated that the key purposes of the lithium studies were to: (1) establish evidence-based dosing strategies for lithium in children and adolescents; (2) characterize the pharmacokinetics and biodisposition of lithium in youth; (3) examine the acute efficacy of lithium in pediatric bipolarity; (4) investigate the long-term effectiveness of lithium treatment; and (5) comprehensively and meticulously characterize the short- and long-term safety of lithium in children and adolescents. RFPs are peer-reviewed and all proposals submitted are scored based upon technical merit in response to the criteria set forth in the RFP. The offerors who submit the proposal with the highest technical score and business proposals are then awarded a contract to perform the clinical studies. The NICHD then submits an Investigational New Drug Application (IND) to the FDA for the proposed studies. The data generated from these trials will be submitted to the FDA and it is anticipated that the label of lithium will be changed to reflect the outcome of these important clinical trials.

## Results

### Submission and Development of Studies

In order to respond to this RFP, the **Co**llaborative **L**ithium **T**rials (**CoLT**) group was formed. The current CoLT team, which is lead by investigators from Case Western Reserve University (P.I. Findling), also includes investigators from Cincinnati Children's Hospital Medical Center/University of Cincinnati (P.I. Kowatch), Cambridge Health Alliance (P.I. Frazier), Children's Hospital & Regional Medical Center Seattle Washington (P.I. McClellan), University of North Carolina (P.I. Sikich), University of Illinois at Chicago (P.I. Pavuluri), and The Feinstein Institute for Medical Research of the North Shore–Long Island Health System (P.I. Kafantaris). These sites were selected specifically based upon the sites' investigators' established scientific expertise in pediatric bipolar disorder as well as clear evidence of being able to consistently, successfully, and safely recruit youths into prospective pediatric bipolar treatment studies. Proposals were submitted for competitive review in April of 2005.

As a result of the CoLT group's submission, these investigators were subsequently awarded this government contract to study lithium in juvenile mania. As part of the work that was to be conducted under the auspices of this contract from the NICHD, collaboration with the Best Pharmaceuticals for Children Act-Coordinating Center (BPCA-CC; Premier Research; Medical Director, B. Brownstein, M.D.) and the CoLT team was established. In addition to the BPCA-CC, the CoLT team also began to collaborate with the NICHD Project Officer (P. Taylor-Zapata, M.D.) in order to propose final study designs to the FDA prior to initiating the requisite clinical trials.

During this protocol refinement process, the CoLT team integrated feedback and input from the NICHD and the BPCA-CC into the study protocols. Although it was originally indicated that three distinct studies were to be performed to meet the goals of the WR, the CoLT team, BPCA-CC, and NICHD collaboratively created two multi-phase trials that would both: (1) ensure that the data that were outlined in the WR were obtained, and (2) allow feasibility of implementation to be enhanced. Each of these two multi-phase studies consists of four phases. The designs of both of these clinical trials were subsequently reviewed by the FDA in February, 2006. Enrollment into the first of these studies began in December of 2006.

### Lithium Formulations and Daily Dosing

It should be noted that throughout these studies, immediate release lithium carbonate will be used due to its availability as a generic formulation. In addition, patients will receive treatment in 300 mg dose increments and for doses of 900 mg or greater, lithium will be given in thrice daily divided doses.

### Ethical Approval and Informed Consent

These studies will be conducted in full accordance with the principles of the Declaration of Helsinki (52nd WMA General Assembly, Edinburgh, Scotland, October 2000). Additionally, prior to enrollment, these studies will be approved by all sites' Institutional Review Board for Human Investigation, and an independent Data Safety Monitoring Board (DSMB) will monitor the studies.

Written informed consent will be acquired from all participants' legal guardians. Additionally, all participating youths will provide written assent prior to the initiation of any study related procedures.

### Inclusion and Exclusion Criteria

Similar entry criteria for each of these outpatient clinical trials will be employed. In short, medically healthy children and adolescents (ages 7–17 years) with bipolar I disorder experiencing a manic or mixed episode may be eligible to enroll. These inclusion and exclusion criteria were developed in order to permit many youths suffering from mania to enroll. However, it was felt that the participation of some youths with selected comorbidities might confound the results of this work. For that reason, a limitation of the CoLT trials is that the data collected may not be applicable to all patients with bipolar I disorder. The inclusion and exclusion criteria for both studies are shown in Tables 1 and 2.

**Table 1:** Inclusion Criteria

**Inclusion Criteria**
1. Subjects aged 7 years to 17 years, 11 months old at time of first dose
2. Patients must meet DSM-IV diagnostic criteria, as assessed by a semi -structured assessment (KSADS-PL) and a separate clinical interview with a child/adolescent psychiatrist for manic or mixed episodes in bipolar I disorder
3. Score of > 20 on the YMRS at screening and baseline
4. The patient and legal guardian must understand the nature of the study and be able to comply with protocol requirements. The legal guardian must give written informed consent and the youth, written assent.
5. Patients with comorbid conditions [attention deficit hyperactivity disorder (ADHD), conduct disorder] may participate.
6. If female: is premenarchal, or is incapable of pregnancy because of a hysterectomy, tubal ligation, or spousal/partner sterility. If sexually active and capable of pregnancy, has been using an acceptable method of contraception (hormonal contraceptives, intrauterine device, spermicide and barrier) for at least one month prior to study entry and agrees to continue to use one of these for the duration of the study. If sexually abstinent and capable of pregnancy, agrees to continued abstinence or to use of an acceptable method of birth control (either intrauterine device or spermicide and barrier) should sexual activity commence
7. Has a negative quantitative serum ß-human chorionic gonadotrophin hormone pregnancy test at screening and a negative qualitative urine pregnancy test at baseline, if female
8. Patients with a history of substance abuse may participate if they agree to abstain from drugs during the trial and have a negative drug screen at screening or prior to baseline.
9. The subject is willing and clinically able to wash out of exclusionary medication during the screening period. Prior to the administration of lithium, patients will not have used any of the following mediations: antipsychotics, monoamine oxidase inhibitors, antidepressants within the preceding two weeks; stimulants within the preceding week; or fluoxetine or depot antipsychotics in the past month (no stable patients will be asked to discontinue medications)
10. ECG and blood work including CBC, prothrombin/partial thromboplastin time, fibrinogen, and thyroid function showing no clinically significant abnormalities

**Table 2:** Exclusion Criteria

Exclusion Criteria
1. Patient who is clinically stable on current medication regimen for bipolar disorder.
2. A current or lifetime diagnosis of Schizophrenia or Schizoaffective Disorder, a Pervasive Developmental Disorder, Anorexia Nervosa, Bulimia Nervosa, or Obsessive-Compulsive Disorder
3. Current DSM-IV diagnosis of Substance Dependence
4. Positive drug screen at screening and on retest 1–3 weeks later
5. Patients with symptoms of mania that may be attributable to a general medical condition, or secondary to use of medications (e.g., corticosteroids)
6. Evidence of any serious and/or unstable neurological illness for which treatment under the auspices of this study would be contra-indicated
7. Any serious, unstable medical illness or clinically significant abnormal laboratory assessments that would adversely impact the scientific interpretability or unduly increase the risks of the protocol
8. Current general medical condition including neurological disease, diabetes mellitus, thyroid dysfunction, or renal dysfunction that might be affected adversely by lithium, could influence the efficacy or safety of lithium, or would complicate interpretation of study results
9. Evidence of current serious homicidal/suicidal ideation such that in the treating physician's opinion it would not be appropriately safe for the subject to participate in this study
10. Evidence of current active hallucinations and delusions such that in the treating physician's opinion it would not be appropriately safe for the subject to participate in this study
11. Concomitant prescription of over-the-counter medication or nutritional supplements that would interact with lithium or the subject's physical or mental status
12. Concurrent psychotherapy treatments provided outside the study initiated within 4 weeks prior to screening
13. Previous adequate trial with lithium (at least 4 weeks with lithium serum levels between 0.8–1.2 mEq/L)
14. History of allergy to lithium
15. Psychiatric hospitalization within 1 month of screening
16. Clinician's judgment that subject is not likely to be able to complete the study as an outpatient due to psychiatric reasons
17. History of lithium intolerance
18. Females who are currently pregnant or lactating
19. Sexually active females who, in the investigators' opinion, are not using an adequate form of birth control.
20. Subjects who are unable to swallow the study medication
21. Subjects for whom a baseline YMRS score of < 20 is anticipated
22. Subjects with an IQ less than 70 (determined using the Wechsler Abbreviated Scales of Intelligence {WASI}Vocabulary and Matrix Reasoning Subscales) [33]

### Overview of Studies 1 and 2

Due to their anticipated sample size of approximately 260 patients and their methodological rigor, when completed, the CoLT studies should provide definitive data about the acute efficacy and long-term treatment with lithium in children and teenagers with bipolar mania/mixed states. A brief description of each of these two studies is presented below.

Study 1 includes an initial open-label phase lasting 8 weeks during which a variety of dosing paradigms will be explored and data for pharmacokinetic analyses will be obtained. This phase is then followed by a 16 week open-label, long-term stabilization phase and a subsequent double-blind discontinuation phase. During this discontinuation phase, eligible patients are randomly assigned to either continue with lithium treatment or receive placebo for up to 28 weeks. Subsequently, the final phase of this first study includes a restabilization phase that allows subjects who suffer a mood symptom relapse during the discontinuation phase to re-initiate open-label lithium therapy. It is planned that this first study will enroll 60 subjects who will receive up to a maximum of 52 weeks of treatment.

Study 2, which will begin subsequent to completion of the first study, begins with an 8-week, double-blind, placebo-controlled phase where 200 subjects will be randomized to receive either lithium or placebo. Similar to the first study, this initial phase will be followed by an open-label long-term phase. However, unlike the analogous phase in the first study, participation in this open-label long term phase in this trial will be of either 16 or 24 weeks in duration depending on the treatment that the patient received during the double-blind phase of this protocol. The long-term treatment phase will be followed by two subsequent phases: a discontinuation phase and a restabilization phase, identical to those described in Study 1. As in Study 1, this study will allow patients to receive treatment with lithium for up to 52 weeks in duration. These studies are described in more detail below. Our hypothesis in Study 1 is that rational dosing strategies for lithium in children and adolescents will be able to be developed. Our hypotheses in Study 2 are (1) that lithium will reduce manic symptoms to a greater extent than placebo acutely and (2) that few participants treated with lithium will withdraw because of adverse effects. Both studies will address the hypotheses (1) that lithium will have long-term efficacy for reducing bipolar symptoms and (2) that lithium will be safe and generally well-tolerated for up to one year.

### Study 1 (see Figure 1)

**Figure 1:**
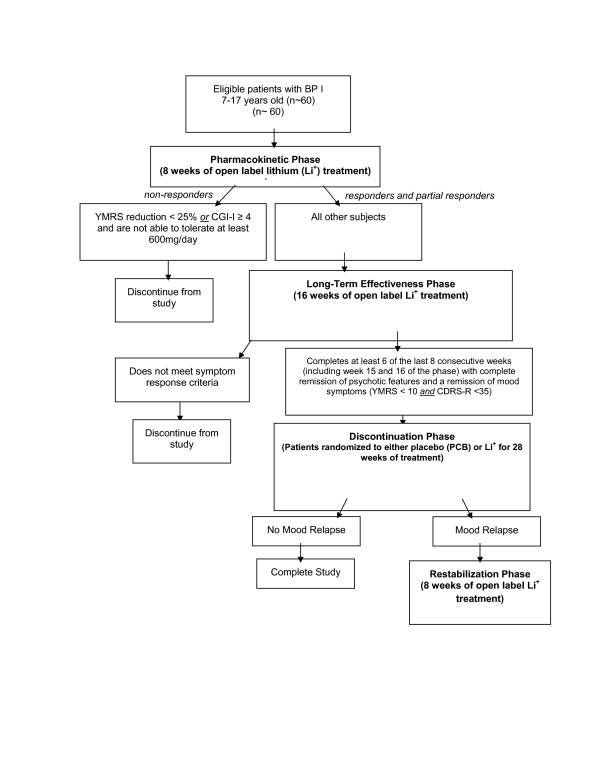
Study 1 of CoLT.

As noted above, Study 1 is comprised of four phases that will address the following key components of the WR and the RFP: (1) establishing evidence-based dosing strategies for lithium in youth; (2) characterizing the pharmacokinetics and biodisposition of lithium in children and adolescents; (3) investigating the long-term effectiveness of lithium treatment; and (4) comprehensively characterizing the short- and long-term safety of lithium in children and adolescents. Patients' continuation into subsequent phases of this study is dependent upon their response to their current treatment in the phase of study in which they are participating. Throughout both studies, the *a priori *response criteria found in Table 3 will be used in order to determine eligibility to participate in subsequent study phases.

**Table 3:** *A priori *response criteria used throughout both studies

**Response Criteria**		
**Non-Response**	**Partial Response**	**Response**

< 25% reduction in baseline YMRS	25–49% reduction in baseline YMRS	≥ 50% reduction in baseline YMRS
OR	AND	AND
CGI-I ≥ 4	CGI-I ≤ 3	CGI-I = 1 or 2

#### Pharmacokinetic Phase

The Pharmacokinetic Phase of Study 1 is an 8-week, open-label, randomized, escalating dose clinical trial that has two key objectives. The first is to characterize the pharmacokinetic profile of lithium. The second is to develop evidence-based dosing strategies for lithium in children and adolescents with bipolar I disorder. Three different starting doses of lithium carbonate will be explored: 300 mg, 600 mg, and 900 mg. Additionally, two different dose escalation strategies will be examined. In one, the dose of lithium will be increased weekly by 300 mg. In the other, the dose of lithium will be increased by 300 mg twice weekly.

In this initial phase of Study 1, subjects will be assigned to one of three treatment arms. A total of approximately 60 subjects, in which approximately 20 subjects will be assigned to each of the three study arms, will be enrolled and dosed. Initially, subjects will be assigned to one of two different treatment groups: "Arm I" or "Arm II".

In Arm I of this initial phase, subjects will be given a starting dose of 300 mg or 600 mg, depending on their weight. All subjects that weigh less than 30 kg will be assigned to Arm I and will receive a starting dose of 300 mg/day. All initial subjects weighing greater than or equal to (≥) 30 kg, after being stratified by age and sex, will be randomly assigned to Arms I and II in approximately equal numbers. All subjects whose weight is ≥ 30 kg and are assigned to Arm I will have a starting dose of 600 mg/day (divided twice daily). In Arm II, subjects who are randomized to this treatment arm will receive a starting dose of 900 mg (divided thrice daily). An evaluation of data collected from subjects who are treated in Arm I and Arm II will provide information about the appropriate starting dose of lithium in children/adolescents. Subjects randomized to Arms I and II may have their dose increased by 300 mg weekly, based upon response and tolerability. After 10 subjects are enrolled in Arm II and have completed the 8 weeks of treatment, and if at least 8 of the first 10 subjects dosed have tolerated the study drug for at least 8 weeks, enrollment into the third arm (Arm III) may begin. Only subjects weighing ≥ 30 kg will be permitted to enter into Arm III. Subjects enrolled into Arm III will have a starting dose of 900 mg, divided thrice daily.

In order to examine a second dose escalation strategy, subjects in Arm III will have their lithium dose increased twice weekly depending upon the effectiveness and tolerability of lithium in this cohort. As a result of this study arm, the speed that the lithium dose may be increased (weekly vs. twice weekly) in children and adolescents will be determined.

Final dosing for subjects will be determined based upon both response and side effect profile for Arms I-III. For the purposes of this study, subjects will continue to have their dose of lithium increased until any of the following criteria are met: (1) a subject meets response criteria (Clinical Global Improvement Scale (CGI-I) [11] of ≤ 2 and ≥ 50% decrease in the Young Mania Rating Scale (YMRS) [12]; (2) the patient experiences side effects that significantly impact functioning; (3) the serum lithium level is > 1.4 mEq/L [13]; or (4) if the dose exceeds 40 mg/kg/day (with the exception of subjects weighing less than 23 kg who may receive up to 900 mg/day). Based upon the Pharmacokinetic Phase, information will be obtained about the most appropriate starting dose and the speed by which the lithium dose can be increased. This dosing strategy will then be employed in the acute randomized controlled trial that is to be performed under the auspices of Study 2 (below).

### Pharmacokinetic Sampling

In addition to these dosing procedures, subjects in Arms I and II will undergo blood sampling procedures in order to characterize first-dose pharmacokinetic (PK) parameters for lithium. Blood samples will be obtained prior to dosing and at 0.5, 1.5, 1, 1.5, 2, 4, 8, 12, and 24 hours post-dose. Additionally, one half of the subjects will provide a single blood sample for PK analyses at 48 hours post-dose, and one half will provide a single blood sample at 72 hours post-dose.

Furthermore, subjects in Arms I and II will have additional PK samples collected at 2 more time points over the next 16 weeks. The time points for these samples to be collected will be determined by random assignment. These additional samples are collected over a 12 hour period, including a 0 and 12 hour sample and 3 randomly assigned additional samples at the following possible time points: 0.5, 1, 1.5, 2, 4, or 8 hours post-dose.

#### Long-Term Effectiveness Phase

Once the Pharmacokinetic Phase has ended, subjects who continue to be eligible and who demonstrate at least a partial response (reduction in YMRS score of ≥ 25% and a CGI-I score ≤ 3) and are able to tolerate at least 600 mg lithium/day will be eligible to continue with their current dose for 16 weeks in a Long-Term Effectiveness Phase (LTE). Following a standardized algorithm, adjunctive medications may be added during this phase. Of note, a maximum of only 2 adjunctive medications are allowed to be prescribed at the same time to study subjects once participation in this study phase begins. Patients who are prescribed other agents besides lithium with therapeutic serum concentration levels will have their medication levels monitored throughout their participation in this study.

The standardized algorithm includes a sequence of medications to treat residual symptoms of psychosis, mania and hypomania, depression, anxiety, and ADHD (prioritized in that order). These treatment algorithms were developed in order to limit variability across subjects and to provide reasonably interpretable preliminary information regarding adjunctive pharmacotherapy in patients treated with lithium. The rationale concerning the choice of adjunctive medications for residual symptom treatment was informed by various adult data in bipolar I and II disorder, as well as limited data that exist in children and adolescents with bipolar disorder (for a review, see Smarty and Findling 2007) [14]. When no adult or juvenile data existed, algorithms were derived by investigators' consensus, based upon their clinical experiences and consideration of which widely used treatments lacked study to support or refute their use.

Psychotic symptoms are to be treated with risperidone, and if needed, followed by a trial of quetiapine, and then aripiprazole. Furthermore, unresponsive manic and hypomanic symptoms will be initially treated with valproate. If the manic or hypomanic symptoms do not respond to valproate, then quetiapine will be started, followed by a trial of aripiprazole.

To address residual depressive symptoms, lamotrigine will be the first line treatment followed by a trial of quetiapine. If the patient is non-responsive to treatment with quetiapine, concomitant treatment with citalopram will be initiated to address the depressive symptoms. Additionally, patients who experience anxiety symptoms will initially be treated with valproate. Subsequently, unresponsive anxiety symptoms will be addressed with concomitant treatment with quetiapine followed by lamotrigine.

Finally, adjunctive ADHD treatment will be begin with a long acting methylphenidate compound. If it is necessary to initiate another ADHD treatment due to unresponsiveness or intolerance to this initial treatment, a long acting mixed amphetamine salt preparation may be started. The final treatment option for comorbid ADHD symptoms is atomoxetine. These adjunctive interventions will provide additional treatment options for youth with BD for which lithium monotherapy does not address residual mood and other psychiatric symptoms.

At the end of this phase, subjects will be categorized as "responders," "partial responders," and "non-responders" based upon *a priori *criteria (Table 3). Subjects who have 6 out of the last 8 consecutive weeks starting at week 8 (the last two weeks must be final 2 weeks of participation in the LTE Phase) without symptom relapse and who have therapeutic lithium levels are eligible for continuation into the Discontinuation Phase.

#### Discontinuation and Restabilization Phases

The third phase, Discontinuation Phase, is a 28-week, double-blind phase where subjects are randomized to receive either continued treatment with lithium or placebo. Subjects who are randomized to receive placebo will have their dose of lithium gradually discontinued. It should be noted that the ethical issues associated with a medication discontinuation paradigm were carefully reviewed. However, in the absence of maintenance treatment data for pediatric bipolar disorder, the investigators believed that the discontinuation phase of the study provided clinical equipoise between the potential risks of side effects related to long-term lithium exposure, and the potential risks for relapse in patients randomized to placebo. While patients are enrolled in Discontinuation Phase, patients may continue to receive the adjunctive medication at the same dose that was prescribed during the Long-Term Effectiveness Phase. During the Discontinuation Phase, if subjects experience a significant deterioration in clinical status, they are offered 8 weeks of open-label lithium treatment re-initiated in a Restabilization Phase.

### Study 2 (see Figure 2)

**Figure 2:**
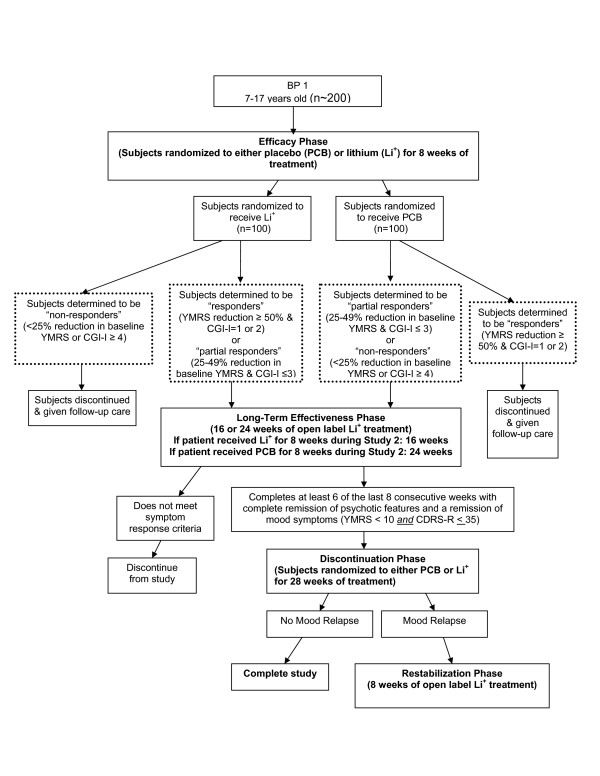
Study 2 of CoLT.

Like Study 1, Study 2 is comprised of four phases that will investigate the acute efficacy of lithium in pediatric bipolarity, examine the long-term effectiveness of lithium treatment, and allow for both the short- and long-term safety of lithium in youth as outlined in the WR. As in Study 1, patients will continue into subsequent phases of the study based upon their response to their response to their current phase of treatment.

#### Efficacy Phase

The first phase of Study 2 is the 8-week, double-blind, parallel-group, placebo-controlled Efficacy Phase. During the Efficacy Phase, approximately 200 subjects will be randomized to receive 8 weeks of treatment with either lithium carbonate or placebo. The starting lithium dose and the rate at which lithium will be titrated upwards will be based upon the results of the Pharmacokinetic Phase in Study 1. At the end of 8 weeks of treatment, response status will be evaluated in all participating subjects.

#### Long-Term Effectiveness Phase

Similar to Study 1, at the end of the first 8 weeks of the study and depending on their response to blinded treatment, the patient may proceed to the Long-Term Effectiveness Phase. However, subjects who are responders to placebo or non-responders to lithium treatment will not continue into the Long-Term Effectiveness Phase. In Study 2, the length of participation in this phase will be dependent upon the treatment and response status of subjects in the Efficacy Phase. This is done in order to ensure all subjects receive open label lithium for 24 weeks prior to possible participation in the Discontinuation Phase. As a result, eligible subjects will continue into two parallel treatment arms of different lengths of time in the Long-Term Effectiveness Phase. The two arms include a 16-week arm and a 24-week arm. Those subjects who received lithium in the Efficacy Phase and showed partial or full response will be eligible to continue in the 16-week arm. During the 16-week arm, adjunctive medications may be added following the standardized algorithm noted above as needed.

Those subjects who received placebo during the Efficacy Phase and are either partial responders or non-responders (YMRS reduction of < 25% or a CGI-I score ≥ 4) will receive open-label lithium treatment in the 24-week treatment arm of the Long-Term Effectiveness Phase. After an initial 8 weeks of treatment with open-label lithium, these subjects will be assessed; if subjects are at least partial responders, they will continue treatment with lithium for the remaining 16 weeks. During the final 16 weeks of the 24-week arm, adjunctive psychotropic agents will be permitted per the aforesaid treatment algorithms. If subjects are non-responders at the end of 8 weeks of treatment in the 24-week arm, they will be discontinued from the study.

Furthermore, subjects who received placebo during the Efficacy Phase will be randomized to possibly receive psychosocial treatment at the onset of the 24-week Long-Term Effectiveness Phase in order to explore whether additional benefit to open-label lithium initiation occurs when combined with psychotherapy. In addition, in order to explore the neurological effects of lithium in juveniles, electro-encephalograms (EEG) will be obtained in the 24-week treatment arm for a randomly chosen subset at baseline, end of week 8, and the end of the Long-Term Effectiveness Phase participation.

As in Study 1, patients will be eligible for participation into the Discontinuation Phase if they have had at least 6 of the last 8 weeks (including the last 2 weeks) of the LTE Phase without symptom relapse and have therapeutic lithium levels.

#### Discontinuation and Restabilization Phases

The Discontinuation and Restabilization phases in Study 2 are identical to these phases in Study 1. Half of the responders will remain on lithium maintenance treatment, and the other half will undergo gradual tapering of their lithium dose by the substitution of placebo capsules for active lithium capsules for 28 weeks. Subjects who experience significant deterioration in clinical status during the Discontinuation Phase will be offered 8 weeks of treatment with open-label lithium in the Restabilization Phase. Data from these final study phases (Discontinuation and Restabilization) will be combined from both Study 1 and Study 2 for statistical analyses.

### Study Teams and Maintaining the Blind

In order to maintain the blind throughout the two trials, two different study teams will be assembled at each of the sites analogous to the study design implemented by the RUPP research group [15]. At each CoLT site, there will be two groups of clinicians and coordinators that compose the "blinded" team and an "unblinded" team. At a minimum, each team will include a child and adolescent psychiatrist and a study coordinator. The blinded study team will manage all aspects of study enrollment with the exception of reviewing lithium levels and making lithium concentration-based dose adjustment decisions during placebo-controlled phases. The unblinded teams will be responsible for reviewing the lithium levels and making dosing adjustments in the blinded phases of the two studies.

### Patient Assessments

As requested by the WR, the YMRS will be the primary outcome measure owing to its ability to detect the effects of medication treatment of mania [16]. Patient diagnoses will be based upon results of the Kiddie Schedule for Affective Disorders and Schizophrenia for School-Age Children-Present and Lifetime Episode (K-SADS-PL) [17]. In addition, the WR indicated that the secondary measures should assess attention-deficit/hyperactivity disorder (ADHD) symptomatology, aggressive behavior, irritability, substance abuse, clinical global improvement, and family, school, peer relationships and quality of life. The measures that will be used to assess these domains during the trials are shown in Table 4.

**Table 4:** Mood Symptomatology and Life Satisfaction Measures obtained in the CoLT trials

**Measure**	**Domain**	**Reference**
**Interview with parents & child/adolescent**		

Young Mania Rating Scale (YMRS)	Manic Symptoms	Young et al. [12]
Children Depression Rating Scale (CDRS-R)	Depression Symptoms	Overholser et al. [34]
Brief Psychiatric Rating Scale – for Children (BPRS-C)	Psychosis	Hughes et al. [35]
Children's Global Assessment Scale (CGAS)	Global Outcome	Shaffer et al. [36]
Clinical Global Impressions Scale–Severity (CGI-S)	Severity of illness	NIMH [11]
Clinical Global Impressions Scale–Improvement (CGI-I)	Improvement of illness	NIMH [11]
Drug Use Severity Inventory (DUSI)	Substance Use	Tarter et al. [37]
Irritability, Depression, and Anxiety (IDA) (selected items)	Irritability	Snaith et al. [38]
The Pediatric Anxiety Rating Scale (PARS)	Anxiety	The Research Units on Pediatric Psychopharmacology Anxiety Study Group [39]
Social Adjustment Inventory for Children & Adolescents (SAICA)	Social Development, Academic Achievement	John et al. [40]
Suicide Severity Rating Scale (SSRS)-Lifetime	Lifetime suicidal ideation and behavior	Posner et al. [41]
Suicide Severity Rating Scale (SSRS)	Suicidal ideation and behavior	Posner et al. [41]

**Parent Report**		

General Behavior Inventory – Parent Report Mania and Depression Short Form	Manic and Depression Symptoms	Youngstrom et al. [42]
AD/HD Rating Scale-IV (ARS-IV)	ADHD symptoms	DuPaul et al. [43]
Child Mania Rating Scale-Parent (CMRS-P)	Manic symptoms	Pavuluri et al. [44]
Nisonger Child Behavior Rating Form (NCBRF) Parent Version	Aggression	Aman et al. [45]
Caregiver Strain Questionnaire (CSQ)	Parental Stress	Brannan et al. [46]
Family Environment Scale (FES)	Family Relationships	Moos & Moos [47]

### Neurocognitve Testing

The WR also required that possible cognitive and neurological effects of lithium be evaluated. Lithium can potentially improve certain cognitive functions, but can be deleterious in other domains. The purpose of this adjunctive testing is to provide an evidence-based understanding of the neurocognitive effects of lithium pre- and post-acute trial and post-maintenance trial. The data collected will provide a comprehensive characterization of lithium-specific effects on neurocognitive function that has not been available to date. Therefore, in Study 2, all subjects will undergo a neurocognitve battery at baseline prior to receiving lithium or placebo, at week 8, and after 24 weeks of lithium treatment.

The goal of this testing is to determine the integrity of fine-motor, attention, verbal memory, and selected executive function domains pre- and post-acute and maintenance lithium trials. It is hypothesized that positive improvement will be noted in domains of attention, verbal memory, visual memory, and selected executive functions (e.g., set-shirting, inhibition) post-treatment. In contrast, based upon data from adult studies, it is hypothesized that lithium may negatively affect fine-motor speed and control and cognitive processing speed, but results may vary based upon response to lithium and serum levels. In addition, testing will help to determine the integrity of affective regulation, including affective inhibition, pre- and post-acute and maintenance trials. It is hypothesized that the affective regulation of this sample will improve from baseline to the proposed post-acute and maintenance trial time points.

Given the available literature on the pathophysiology of bipolar disorder, these assessment domains were selected to coincide with brain regions where the effects of this disorder would be most expected to occur (i.e., hippocampal and pre/frontal brain regions). In addition, tasks were selected to: (1) be age-appropriate and child friendly; (2) have adequate statistical applicability to the various ages and ability levels of this population; (3) be psychometrically sound (i.e., reliable, valid); and (4) theoretically driven by the available literature that has examined lithium usage in children and adults, as well as the extant literature on pediatric bipolar disorder. Further, given the 8-week differential between some of the tasks, measures that evidenced minimal practice effects over this time frame were selected. The neurocognitive tests that will be used in this trial are shown in Table 5.

**Table 5:** Neurocognitive measures to be collected in the CoLT study

Domain					
Intellectual	Fine Motor	Attention	Memory	Executive	Affective Processing

WASI 2 Subtest (WASI) [33]	Grooved Pegboard [48-50]	Vigil Auditory CPT [53]	WRAML-2 Verbal Memory (2 Subtests) [54]	D-KEF Verbal Fluency (Conditions 1–3) [51,52]	Affective Stroop Task [55]
	Delis-Kaplan Executive Function System	Vigil Visual CPT [53]	WRAML-2 Delayed Verbal Memory [54]	D-KEF Figural Fluency (Condition 1) [51,52]	Affective N Back Memory Task [56]
	(D-KEFS) Trail Making (Condition 4) [51,52]			D-KEF Color-Word [51,52]	

### Safety Assessments

#### Adverse Event Monitoring

Subjects and their guardians will be directly queried about the presence of adverse events throughout the CoLT trials. In addition, to facilitate the careful monitoring of adverse events, multiple assessments will be utilized throughout both studies. These include the Neurological Examination for Lithium (NELi), a modified Side Effects Form for Children and Adolescents (SEFCA) [18], and the Neurological Rating Scale (NRS) [19].

The NeLi, which was developed specifically for this trial, includes an examination of neurological events that have been associated with Li treatment. These neurological symptoms include: (1) Tremor; (2) a Finger-nose Test; (3) Tandem Walk; (4) Gait; (5) Grip Strength; and (6) the Romberg Test.

The SEFCA is a 54-item scale that rates both the frequency and severity of adverse events. In addition, the SEFCA used in the CoLT studies will be supplemented by selected UKU Side Effect Rating Scale [20] items including queries regarding concentration difficulties, increased fatigability, sleepiness/sedation, reduced salivation, and memory difficulties. Furthermore, items that pertain to acne, motor in-coordination, muscle weakness, and confusion will be added to the SEFCA from the Safety Monitoring and Uniform Report Form (SMURF) [21].

#### Laboratory and Electrocardiogram (ECG) Monitoring

Over the course of the CoLT trials, laboratory and ECG testing will be performed periodically. The chemistry profile that will be used throughout the CoLT trials will measure blood concentrations of sodium, potassium, chlorine, carbon dioxide, blood urea nitrogen (BUN), creatinine, calcium and glucose. Furthermore, blood concentrations of total protein, albumin, alkaline phosphate, alkaline transferase, alkaline aspartate, and total bilirubin will be obtained.

To study prospectively the effects of lithium on metabolism and lipid profile, fasting total cholesterol, triglycerides, high density lipo-proteins (HDL), low density lipo-proteins (LDL), and cholesterol/HDL ratio will be acquired on all subjects at specified times during the CoLT trials. Additionally, a Complete Blood Count (CBC) with differential will be performed periodically. A urinalysis and urine drug toxicology screen will be assessed at various time points during both CoLT trials.

Lithium has been found to interfere with the production of thyroid hormones including the inhibition of the thyroid stimulating hormone (TSH)-responsive adenylate cyclase and PKC in thyroid cells [22-24]. Therefore, thyroid function tests, including TSH (thyroid stimulating hormone), triiodothyronine, and thyroxine will be regularly obtained in theses studies. It should be noted that the CoLT trials also incorporate an algorithm for the assessment and management of TSH elevation/hypothyroidism should either event occur during the auspices of these trials.

In addition, research indicates that about 5% of subjects treated with lithium may develop kidney dysfunction as indicated by impaired renal function tests [25]. For this reason, creatinine clearance will be measured at various time points throughout the CoLT trials in order to further assess renal function.

#### Lithium Serum Levels

Monitoring lithium serum concentrations is critical for the safe use of this agent. It has been suggested that the therapeutic serum concentration range for treatment with lithium lies between 0.3 and 1.3 mEq/L, with 1.5 mEq/L representing the lower limit for intoxication [13]. Additionally, it has been recognized that lithium has a narrow therapeutic index and near-toxic doses are required to achieve the optimal therapeutic effect [26,27]. Therefore, the chosen maximum lithium level after which dose increases would not be permitted was set at 1.4 mEq/L. Lithium serum concentrations will be obtained weekly during the first 8 weeks of treatment and will be monitored regularly thereafter.

#### Electrocardiogram (ECG)

The cardiovascular effects of orally administered lithium have been reported as being generally benign. However, lithium has been shown to prolong sinus node recovery time [28,29]. Therefore, an ECG will be utilized prior to patients receiving lithium and throughout the CoLT trials in order to monitor cardiac function.

#### Electroencephalogram (EEG)

As stated above, an EEG will be obtained from a randomly assigned subset of subjects who participate in the 24-week long Long-Term Effectiveness Phase at baseline, at the end of week 8, and at the 24-week time point. In addition, if a subject experiences significant deterioration in neurological or cognitive status, an EEG will also be obtained. Furthermore, if a patient experiences moderate or severe headaches that are temporally related to medication/placebo, dysarthria, ataxia, cognitive dulling, or confusion that are possibly or probably related to the study medication an EEG will be obtained.

#### RBC/Plasma Lithium (Li^+^) Ratio

RBC/plasma Li^+ ^ratio is considered to have clinical implications in: (1) predicting clinical response; (2) risk for toxicity; (3) possibly, time course for response; and (4) optimal dosage for long-term prophylaxis. Researchers have found that high *in vivo *RBC/plasma Li^+ ^ratios are a result of a RBC membrane defect that causes a deficiency of Li^+ ^- Na^+ ^counter flow [30]. In addition, there is preliminary evidence that this membrane defect is autosomal dominant in transmission, and leads to high intracellular Li^+ ^level compared to serum level [30,31]. A high Li^+ ^ratio is considered to be predictive of a positive lithium response [32].

Therefore, during the 24-week Long-Term Effectiveness arm, a subset of 24 randomly subjects will have weekly serum and whole blood samples to allow for a Li^+ ^ratio to be computed. In addition, subjects who experience ataxia, dysarthria, reduced motor coordination, listlessness/sedation, slurred speech, tremors, confusion, or delirium that is both possibly or probably related to the study medication, and is noted to be of moderate or severe intensity, will have a serum and a whole blood sample in order to assess the RBC/plasma Li^+ ^ratios.

Throughout the trials, a Data Safety Monitoring Board (DSMB) will be involved in monitoring the trials' progress.

## Conclusion

In summary, the innovative and multidisciplinary CoLT studies will provide the data to allow for evidence based dosing of lithium in the children and adolescents with bipolar disorder. In addition, if lithium is shown to have an acceptable acute- and long-term efficacy and safety profile in children and adolescents, this knowledge could substantively influence the treatment choices of clinicians who provide care to those vulnerable children and teenagers suffering from bipolar disorder.

## Competing interests

Dr. Findling receives or has received research support, acted as a consultant and/or served on a speaker's bureau for Abbott, Addrenex, AstraZeneca, Bristol-Myers Squibb, Forest, GlaxoSmithKline, Johnson & Johnson, Lilly, Neuropharm, Novartis, Organon, Otsuka, Pfizer, Sanofi-Aventis, Sepracore, Shire, Solvay, Supernus Pharmaceuticals, and Wyeth. Dr. Frazier receives or has received research support from Bristol Myers Squibb, GlaxoSmithKline, Eli Lilly and Company, Johnson and Johnson, Neuropharm, Otsuka, and Pfizer. Dr. Kowatch receives or has received research support, acted as a consultant, served on an advisory board, and/or served on a speaker's bureau for Abbott, Astra-Zeneca, Bristol-Myers Squibb, CABF, Creative Educational Concepts, GlaxoSmithKline, Medscape, NICHD, NIMH, Sanofi-Aventis, and the Stanley Research Foundation. Dr. Pavuluri's work unrelated to this manuscript is supported by NIH/NCRR K23 RR018638-01, NIMH MH077852, NIMH P50 HD055751, DANA Foundation, NARSAD, American Foundation for Suicide Prevention, Colbeth Foundation, GlaxoSmithKline- NeuroHealth, Abbott Pharmaceuticals and Janssen Research Foundation. Dr. Sikich receives or has received research support from Eli Lilly, Janssen, Pfizer, Bristol Myers Squibb, Otsuka and Neuropharm. Dr. Hooper has acted as a consultant to Lilly. Dr. Taylor-Zapata is the project officer for the funding institute, the Eunice Kennedy Shriver National Institute of Child Health and Human Development (NICHD), for this study. The other authors have no financial ties to disclose.

## Authors' contributions

All authors have made substantial contribution to the conception and design of the study, have been involved in the drafting and/or critical revising of this manuscript, and all authors have given final approval of this manuscript.
